# Age at onset and gene variants predict lifespan and disease duration in childhood neuronal ceroid lipofuscinoses

**DOI:** 10.1111/dmcn.16416

**Published:** 2025-07-24

**Authors:** Alessandro Simonati, Francesco Pezzini, Nardo Nardocci, CLNet Consortium, C Aiello, R Battini, R Biancheri, M Bonsignore, R Carrozzo, B Dalla Bernardina, S Della Vecchia, S Doccini, A Donati, M Filocamo, B Garavaglia, T Granata, S Grosso, AM Laverda, V Leuzzi, C Marini, M Mastrangelo, T Messena, M Salandin, S Sartori, N Specchio, P Striano, F Taioli, A Tessa, E Veneselli, Filippo M. Santorelli

**Affiliations:** ^1^ Department of Surgery, Dentistry, Paediatrics and Gynaecology University of Verona Verona Italy; ^2^ Neurological Institute C Besta Foundation Milan Italy; ^3^ Molecular Medicine for Neurodegenerative and Neuromuscular Diseases Unit IRCCS Stella Maris Foundation Calambrone (Pisa) Italy

## Abstract

**Aim:**

To address disease progression in a cohort of patients with childhood‐onset neuronal ceroid lipofuscinosis (NCL), a group of genetic disorders leading to progressive dementia.

**Method:**

In this retrospective study, selected clinical features (age at onset, at death, and disease duration) and pathogenicity of individual genotypes were selected and matched from the database of the Italian network of NCLs. A cohort of 152 children with molecularly diagnosed NCL were grouped by age at onset and subdivided according to the associated mutated gene. Clinical features were assessed by descriptive statistics and the significance level by non‐parametric tests. The pathogenicity of patients' genotypes in each NCL form was categorized following the guidelines of the American College of Medical Genetics (ACMG) and matched with the phenotypes.

**Results:**

The median age at onset was 4 years; the median age at death was 17 years. The earliest disease onset was related to the shortest lifespan (6 years). Earlier onset and short survival were associated with mutated genes encoding for lysosomal enzymes. High percentages of pathogenic variants were identified and associated with 79.3% of genotypes. The evaluated clinical parameters were not necessarily linked to the genotype.

**Interpretation:**

The age of onset is a good indicator of the expected lifespan of a child with NCL. The ACMG classes of variant partly foresee the outcome of NCL phenotypes.

## Graphical abstract

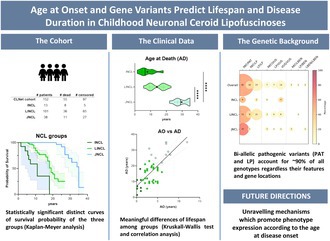

**This original article is commented on by Mole on pages** 156–157 **of this issue.**

**Abbreviations**
ACMG: American College of Medical Genetics
CLNet: Italian network of neuronal ceroid lipofuscinoses
INCL: infantile neuronal ceroid lipofuscinosis
JNCL: juvenile neuronal ceroid lipofuscinosis
LINCL: late infantile neuronal ceroid lipofuscinosis
NCL: neuronal ceroid lipofuscinosis
VUS: variant of uncertain significance

**What this paper adds**

- Cumulative data show a strong correlation between age at onset and lifespan in neuronal ceroid lipofuscinosis.
- The shapes of survival curve decline were parallel among three age‐related groups.
- Lifespan is not influenced by the mutated gene.
- Phenotype severity is unrelated to genotype profiles categorized according to American College of Medical Genetics guidelines.

Neuronal ceroid lipofuscinoses (NCLs), a group of genetically determined diseases, are the most common neurodegenerative disorder in childhood, and one of the main causes of childhood dementia worldwide. All NCLs share common clinical features, have a progressive course, and a fatal outcome. Epidemiological studies have shown some differences of prevalence among the forms.^1^, ^2^, ^3^ At present, NCL nomenclature is based on the genes harbouring variations, and 13 genetic forms are recognized; nine NCLs have childhood onset and are inherited with autosomal recessive trait.^4^, ^5^ NCL genes encode proteins of the lysosomal compartment, the endoplasmic reticulum, and one gene product is associated with cellular membranes.^6^, ^7^ Phenotypic heterogeneity is quite common among the NCLs, possibly associated with a wide spectrum of variation in the same gene having severe or milder clinical consequences.^8^

In this retrospective study we examined disease progression in a cohort of patients with different NCLs of childhood onset. We aimed to measure the patients' lifespan and disease duration, and assess the role of age at onset on determining these. The children were grouped on the basis of age at presentation according to an axial diagnostic system of classification for NCL disorders.^9^ Furthermore, the temporal evolution of the disease and outcome were matched with the patients' genotypes and predicted effects on protein function. Focusing on three age‐related NCL groups, we specifically tested whether the mutation classes of individual variants (categorized according to the guidelines of the American College of Medical Genetics [ACMG]^10^) could have an impact on disease duration and age at death.

## METHOD

### Participants

The cohort of patients was selected from the database of the Italian network of NCLs (CLNet), which collects, in 14 third‐level centres, the clinical, biochemical, molecular, and pathological features of the vast majority of Italian patients with childhood‐onset NCLs, whose data were referred to the database. The diagnostic procedures were performed with parental informed consent and diagnoses obtained in accordance with the Code of Ethics of the World Medical Association (Declaration of Helsinki). The study was approved by the paediatric ethics committee of Regione Toscana (protocol ID 149/2020).

We performed a retrospective analysis of the patients recorded in the CLNet database in December 2021. Patients with childhood‐onset NCL, whose clinical diagnosis was supported by a positive molecular test, were considered in this study (CLNet cohort). Axis 4 of the diagnostic system of classification for NCLs refers to the clinical phenotype^9^ and includes the age at onset: congenital NCL, presenting symptoms at birth, infantile NCL (INCL) starting before 18 months of age, late infantile NCL (LINCL) beginning between 18 months and 6 years, juvenile NCL (JNCL) beginning at early primary school age, late juvenile variants starting at puberty and early adolescence, and adult‐onset NCL. In each age group, patients with available age at onset and eventually age at death were then subdivided according to their genetic classification. Classic CLN1, CLN2, and CLN3 diseases were considered as the representative form of each group; the remaining forms were grouped as NCL variants for each group. None of the patients underwent enzyme replacement or gene therapy protocols. The age at onset was defined as the age at first reported symptoms related to the disease. We obtained the disease duration in patients whose age at onset and age at death were known; moreover, we included ‘censored’ patients whose age at death was not known, but whose disease duration at the last reported clinical consultation was higher than the mean duration minus 1 standard deviation of the patients belonging to the same NCL group. Age at onset, disease duration, age at death, and age at last consultation were considered as the clinical data in this study.

### Genetics

Molecular genetic analyses of all patients were performed by single‐gene sequencing, or by next‐generation sequencing using targeted gene panels of all known NCL genes and exomes according to established protocols. The description of gene variants and predicted protein changes followed the Human Genome Structural Variation Consortium nomenclature (https://www.hgvs.org/). Identified variants were inquired using the most recently released version of the ClinVar database (https://www.ncbi.nlm.nih.gov/clinvar/), the Varsome database (https://varsome.com, release 11.8), and the Franklin database (https://franklin.genoox.com/clinical‐db/home). The Varsome database was used to categorize variants and score their pathogenicity according to the established ACMG guidelines.^10^, ^11^, ^12^ PS3 code was used when activity of lysosomal enzymes was severely reduced and/or nil. ACMG classification spans from class 1 to 5, as benign, likely benign, variant of uncertain significance (VUS), likely pathogenic, and pathogenic respectively.

Gene variants and the corresponding protein changes were both matched with the most recent versions of the NCL mutation database (https://www.ucl.ac.uk/ncl‐disease/mutation‐and‐patient‐database) to check the functional effects of each change (data not shown). Bubble chart representations were used to depict the distribution of variants and genotypes, categorized according either to the type of mutation or to the ACMG pathogenetic scoring system in the NCL groups and forms.

### Statistics

Standard descriptive parameters (mean, standard deviation [SD], median, and interquartile range [IQR]) were used to evaluate the ‘clinical data’ whose distribution and density were represented by violin plots. Non‐parametric tests were applied to assess the difference between two (Mann–Whitney *U* test) or more (Kruskal–Wallis test followed by Dunn's multiple comparisons test) groups of patients. Correlation analysis among different variables was performed by the non‐parametric Spearman method. To assess clinical outcome, Kaplan–Meier survival curves were generated considering either the age at death or the age of last consultation for censored patients; to assess the statistical difference, a log‐rank test was used then *p*‐values were adjusted for multiple comparison following Bonferroni post‐hoc correction. The statistical analyses and graphical representations (including violin plots, survival curves, and bubble charts) were performed using Prism 10.0.2 (GraphPad Software, Boston, MA, USA). A description of methodologies is also reported in Appendix S1.

## RESULTS

### General data

#### Demographic findings

The CLNet cohort included 152 patients belonging to 127 families. Most of them were born from parents of Italian ancestry. The male:female ratio was 1:1 (Table S1). The data confirmed a previous report on the prevalence of NCL forms in Italy.^2^

#### Clinical features

**FIGURE 1:**
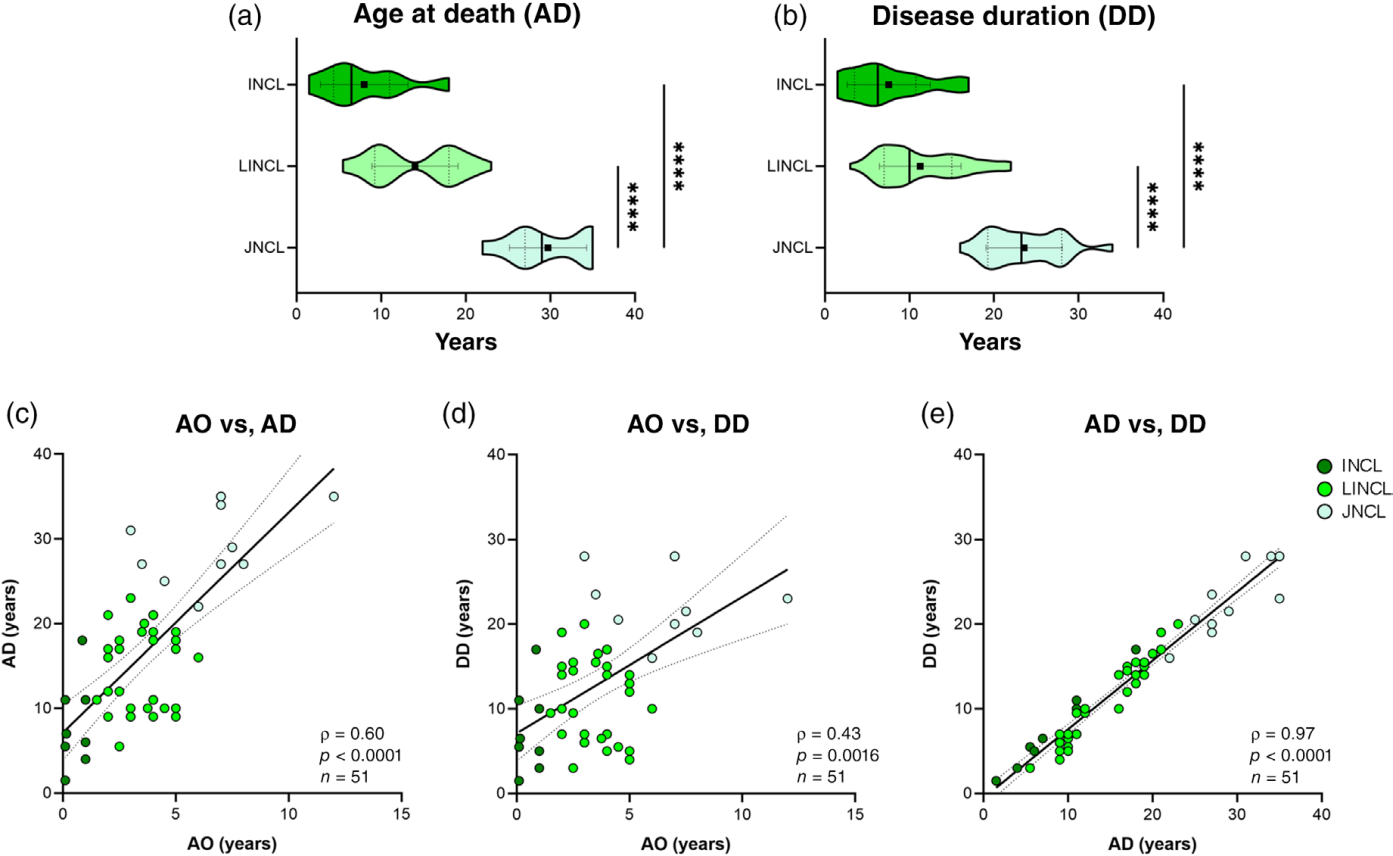
Clinical parameters of the CLNet cohort and related correlation analysis. (a) Violin plots of age at death of CLNet patients (*n* = 55). The INCL group (*n* = 8) showed the lowest mean and median age at death (AD). Two bellies were clearly visible in the plot for the LINCL group (*n* = 36), which corresponded to patients with CLN2 (left) and vLINCL (right). The eldest age at death was for the JNCL group (*n* = 11), whose mean was significantly higher than both the INCL and LINCL groups. (b) Violin plots of disease duration (DD) for both patients and censored patients (*n* = 107) in the cohort. The INCL group (*n* = 12) showed the lowest disease duration values. The LINCL group (*n* = 75) showed a left belly, enclosing almost all patients with CLN2, whereas the remaining ones spanned a longer period. Prolonged disease duration was associated with the JNCL group (*n* = 20). Meaningful statistical differences were measured for both age at death and disease duration between JNCL versus INCL and JNCL versus LINCL groups. Kruskal–Wallis test followed by Dunn's multiple comparisons test; ****p < 0.0001. Black squares and horizontal lines represented mean and SD, whereas median and IQR are depicted as a bold black line and dotted lines respectively. (c–e) Spearman's correlation analysis of CLNet cohort groups (*n* = 51). (c) Correlation analysis revealed a strong, highly significant correlation between the age at onset and age at death for the patients with NCL (*n* = 51) (*ρ* coefficient = 0.60, *p* < 0.0001. (d) Likewise, a moderate but still significant correlation was observed between age at onset (AO) and disease duration in the same groups of patients with NCL (*ρ* coefficient = 0.43, *p* = 0.0016. (e) The age at death and disease duration were also highly correlated (*ρ* coefficient = 0.97, *p* < 0.0001). Patients with LINCL were distributed in two separated groups: one characterized by early age at death (from 5 years 6 months–12 years), the other showing later age at death (from 16–23 years); the first group was formed by patients with CLN2 (*n* = 10) and vLINCL (*n* = 5), whereas patients with vLINCL only (*n* = 17) belonged to the second group. Abbreviations: CLNet, Italian network of neuronal ceroid lipofuscinoses; INCL, infantile neuronal ceroid lipofuscinosis; JNCL, juvenile neuronal ceroid lipofuscinosis; LINCL, late infantile neuronal ceroid lipofuscinosis; NCL, neuronal ceroid lipofuscinosis.

The age at onset was known in 143 patients, with a median of 4 years (IQR 2 years 6 months–5 years) in the aggregated groups, whereas median age at death (*n* = 55) was 17 years (IQR 9–21 years). The median age at death of the JNCL group was twofold higher than of patients with LINCL and about fourfold higher than those with INCL. The median disease duration (*n* = 51) of the cohort was 13 years (IQR 6 years 6 months–17 years) and 11 years (IQR 7–19 years) including censored patients (*n* = 107). In patients with JNCL, the disease duration was longer: fourfold higher than the infantile and twofold higher than late infantile groups. Overall clinical data are described in Table S2 and summarized in Table 1. Age at death and disease duration are depicted as violin plots; significant statistical correlations between the clinical data are also represented (Figure 1).

**TABLE 1:** Clinical data of the CLNet cohort.

		Age at onset, years:months			Age at death, years:months			Disease duration^a^, years:months		
	Overall *n*	*n*	Mean (SD)	Median (IQR)	*n*	Mean (SD)	Median (IQR)	*n*	Mean (SD)	Median (IQR)
**CLNet cohort**	152	143	4:2 (2:7)	4 (2:6–5:0)	55	16:4 (8:7)	17:0 (9:0–21:0)	107	13:2 (7:0)	11:0 (7:0–19:0)
**INCL**	13	12	0:8 (0:5)	0:11 (0:1–1:0)	8	8:0 (5:2)	6:6 (4:5–11:0)	12	7:6 (4:11)	6:4 (3:6–10:10)
CLN1	7	7	0:11 (0:1)	1:0 (0:11–1:0)	4	9:10 (6:2)	8:6 (4:6–16:4)	7	8:7 (5:10)	8:0 (3:0–15:0)
vINCL	6	5	0:4 (0:4)	0:1 (0:1–0:6)	4	6:4 (3:11)	6:4 (2:6–10:0)	5	6:1 (3:5)	6:0 (3:6–8:10)
**LINCL**	101	95	3:6 (1:2)	3:6 (2:6–4:0)	36	14:0 (5:1)	14:0 (9:4–18:0)	75	11:4 (4:10)	10:0 (7:0–15:0)
CLN2	28	23	2:8 (1:0)	3:0 (2:0–3:10)	13	10:2 (2:10)	9:0 (9:0–11:0)	19	8:4 (3:5)	7:0 (6:0–9:6)
vLINCL	73	72	3:8 (1:2)	4:0 (2:6–4:6)	23	16:2 (4:10)	18:0 (12:0–19:0)	56	12:4 (4:11)	11:6 (8:1–15:11)
**JNCL**	38	36	7:5 (2:7)	7:0 (6:0–8:10)	11	29:8 (4:7)	29:0 (27:0–35:0)	20	23:7 (4:6)	23:4 (19:4–28:0)
CLN3	24	22	6:5 (1:8)	6:6 (5:5–8:0)	8	29:10 (4:11)	30:0 (25:6–34:10)	13	23:2 (4:0)	23:6 (19:10–28:0)
vJNCL	14	14	8:11 (3:1)	8:6 (6:11–12:0)	3	29:8 (4:7)	27:0 (27:0–35:0)	7	24:1 (5:7)	23:0 (19:0–28:0)

^a^ Disease duration includes dead patients with a definite disease duration (*n* = 51) and censored patients (*n* = 57).

Abbreviations: CLN1, ‘classic’ infantile neuronal ceroid lipofuscinosis form; CLN2, ‘classic’ late infantile neuronal ceroid lipofuscinosis form; CLN3, ‘classic’ juvenile neuronal ceroid lipofuscinosis form; CLNet, Italian network of neuronal ceroid lipofuscinoses; INCL, infantile neuronal ceroid lipofuscinosis; IQR, interquartile range; JNCL, juvenile neuronal ceroid lipofuscinosis; LINCL, late infantile neuronal ceroid lipofuscinosis; SD, standard deviation; vINCL, variant infantile neuronal ceroid lipofuscinosis forms; vJNCL, variant juvenile neuronal ceroid lipofuscinosis forms; vLINCL, variant late infantile neuronal ceroid lipofuscinosis forms.

**FIGURE 2:**
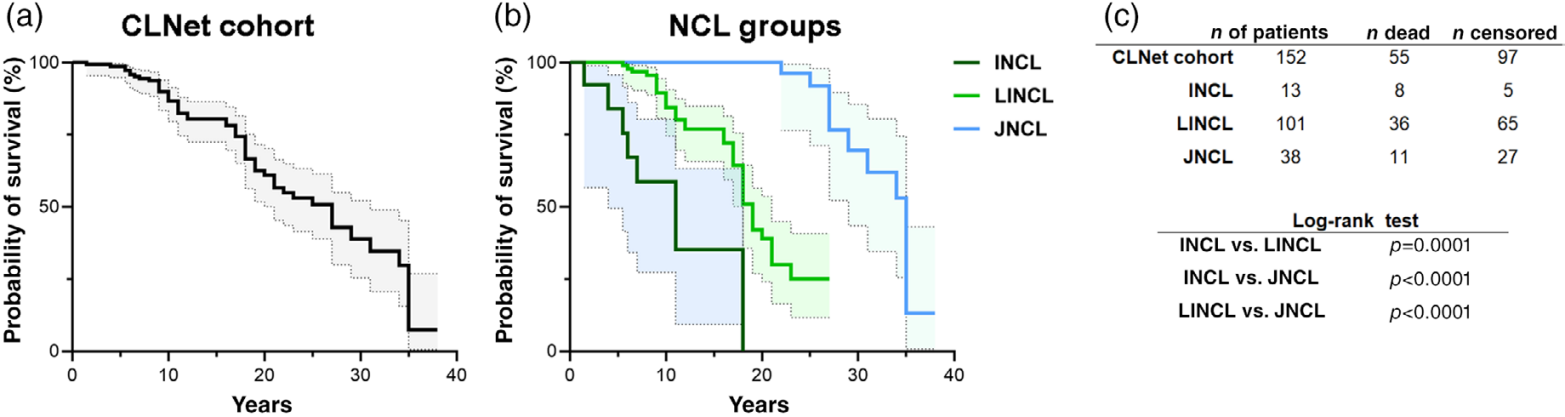
Kaplan–Meier survival curves of all patients in the CLNet cohort and related NCL groups. (a) Considering the entire CLNet cohort (*n* = 152 patients), the Kaplan–Meier analysis indicated that the survival probability starts to decrease at about 5 years of age, reaching 50% around 26 years. (b) The three NCL groups showed distinct survival curves. A very rapid decrease in survival rate was related to INCL, with no patients alive after 18 years of age. The LINCL curve was shifted to right, pinpointing an approximate 30% survival probability after 20 years of age. Patients with JNCL had the curve that was most shifted to the right, which began to fold downwards at around 22 years of age and indicating a 50% survival rate around 35 years. It was noteworthy that the decreasing pattern was similar for the three curves, even if starting at different ages. (c) The numbers of patients used to draw both curves. To assess the statistical differences, a Log‐rank test was used for paired analysis of two curves; then, *p*‐values were adjusted for multiple comparison following Bonferroni post‐hoc correction (*n* = 3 comparisons, corresponding to a Bonferroni‐corrected *α*‐threshold of 0.016). Abbreviations: CLNet, Italian network of neuronal ceroid lipofuscinoses; INCL, infantile neuronal ceroid lipofuscinosis; JNCL, juvenile neuronal ceroid lipofuscinosis; LINCL, late infantile neuronal ceroid lipofuscinosis; NCL, neuronal ceroid lipofuscinosis.

**FIGURE 3:**
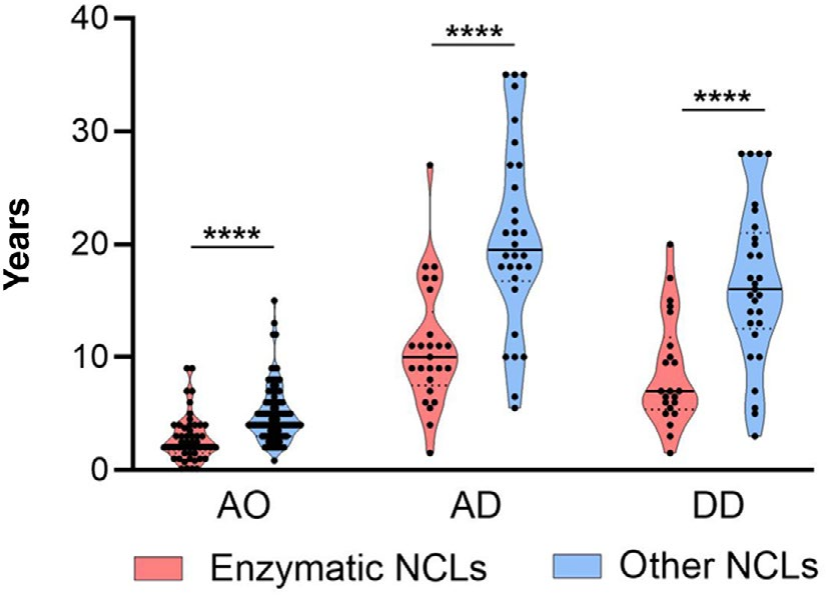
Violin representation of the distribution of the cohort of patients according to the clinical parameters and characterization of the gene products, either mutated primary lysosomal enzymes (in pink) or proteins of other cell compartments (in blue). Patients who carried mutations in lysosomal enzymes had highly significant earlier disease onset (*n* = 50), earlier age at death (*n* = 25), and shorter disease duration (*n* = 22) than those who were carrying mutations of other NCL‐related genes (CLN3, CLN5, CLN6, CLN7, CLN8; *n* = 93, *n* = 30, *n* = 29, and *n* = 64 for age at onset [AO], age at death [AD], and disease duration [DD] respectively). Dots in the upper parts of the age at death and disease duration violins represent patients with variant juvenile NCL forms (see text). Mann–Whitney *U* test; ****p < 0.0001. Black horizontal lines and dotted lines represent median and IQR respectively. Abbreviation: NCL, neuronal ceroid lipofuscinosis.

The Kaplan–Meier survival curve showed a relatively homogeneous trend, revealing 50% of survival probability at 26 years of age. Distinct patterns were observed when the three groups were analysed separately. There was a low survival probability early after disease onset (at about 5–10 years of age) for both the INCL and LINCL groups; conversely patients with JNCL had high chances of longer survival (Figure 2). Cumulative data revealed earlier onset and death of the NCL forms associated primarily with defective lysosomal hydrolases compared with those whose gene products exert different cellular functions in other cell compartments (Figure 3).

### Groups and NCL forms

#### INCL group

**FIGURE 4:**
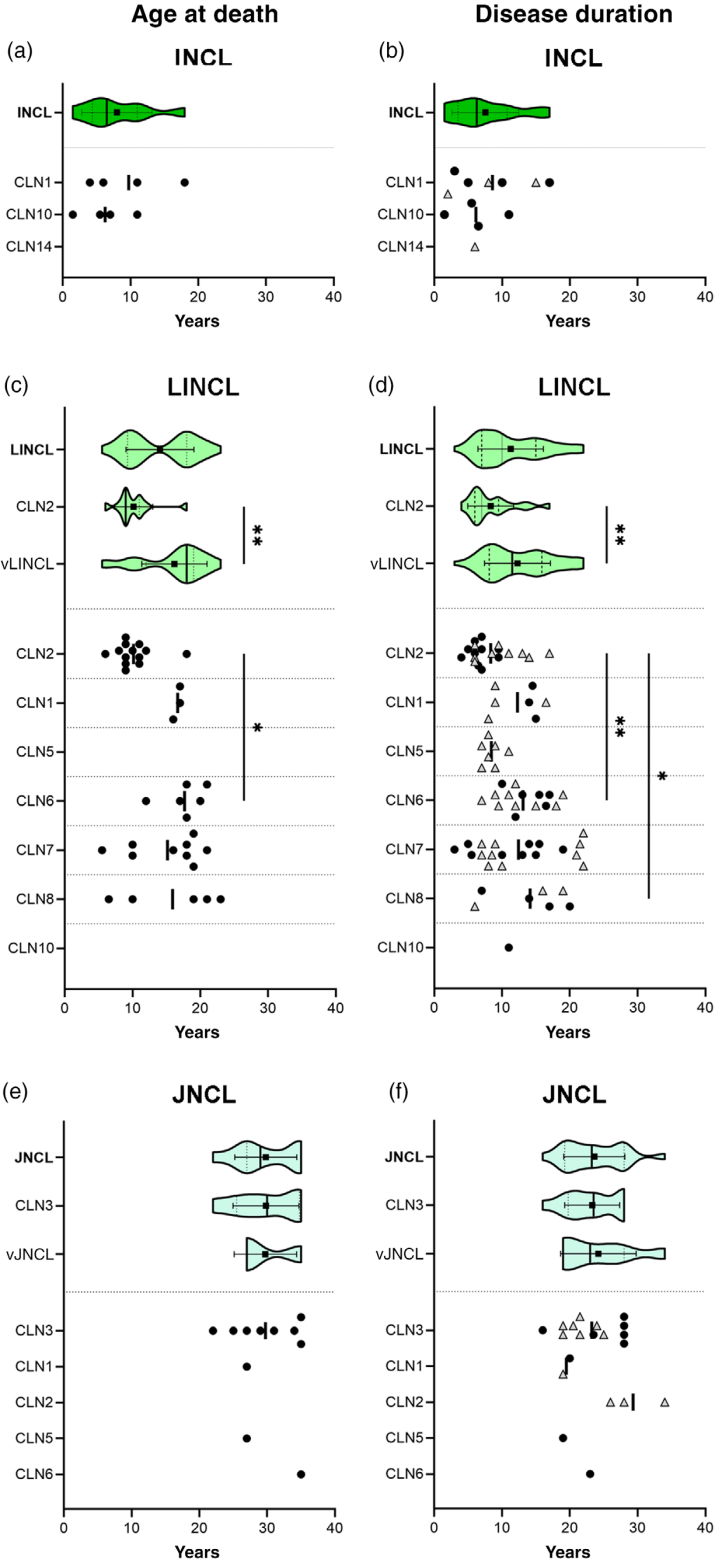
Violin plots representing the distribution of age at death and disease duration for patients with INCL, LINCL and JNCL. (a) Age at death of patients with CLN1 and I‐CLN10 almost overlapped; note the presence of one early outlier in I‐CLN10 (1‐year‐old child) and one late outlier in CLN1 (18‐year‐old child). (b) Disease duration ranged from approximately 1 year for a child with I‐CLN10 to 17 years for the patient with CLN1 who died at the age of 18. The child with I‐CLN14 was included as a censored patient with INCL. (c) The bimodal pattern of violin plots for age at death was associated with patients who had CLN2 (*n* = 13; left belly) and vLINCL (*n* = 23; right belly); the mean ages at death of the two subgroups are statistically different (*p* < 0.001). The distribution of age at death of children with CLN2 was very narrow: only one patient with CLN2 died at 18 years of age. Conversely, a longer lifespan was evident for patients with vLINCL: similar mean and median values for age at death were observed among the different forms (see Table S2). A significant difference between means for age at death of patients with CLN2 and LI‐CLN6 is shown (*p* < 0.05). (d) The violin plot representing the disease duration for all patients with LINCL (*n* = 75) shows a unimodal pattern associated with a long tail of decreasing thickness. Following the separation into the two groups, the distribution of disease duration for patients with CLN2 (*n* = 19) and vLINCL (*n* = 56) was statistically different (*p* < 0.001). A large variability of disease duration was observed for children with vLINCL, mostly patients with LI‐CLN7. The mean disease durations of patients with LI‐CLN6 and LI‐CLN8 were significantly different from that of children with CLN2 (*p* < 0.001 and *p* < 0.05 respectively). Kruskal–Wallis test followed by Dunn's multiple comparisons test; *p < 0.05, **p < 0.001. (e) The violin plots of age at death for patients with CLN3 (*n* = 8) and vJNCL (*n* = 3) overlap, and are distributed over a large time span (below and above 30 years of age). (f) The features of the violin plot representing the disease duration for patients with CLN3 (*n* = 13) were similar to those of patients with vJNCL (*n* = 7). Three censored patients with vJ‐CLN2 showed longer disease durations than the other patients with vJNCL. Black squares and horizontal lines represent mean and SD, whereas median and IQR are depicted as vertical bold black lines and dotted lines respectively. Each child is shown as a black dot, whereas grey triangles in (b, d, and f) indicate censored children. Abbreviations: INCL, infantile neuronal ceroid lipofuscinosis; JNCL, juvenile neuronal ceroid lipofuscinosis; LINCL, late infantile neuronal ceroid lipofuscinosis; vJNCL, variant juvenile neuronal ceroid lipofuscinosis forms.

A small sample of children was collected in this group (*n* = 13), diagnosed with CLN1, I‐CLN10, and I‐CLN14 diseases. This group was characterized by the earliest onset (median age at onset 11 months) and the shortest lifespan (median age at death 6 years and 6 months) in the whole cohort. The disease had a very early onset (in first 2 months of life) in the children harbouring variants in the *CLN10* gene, whereas it started later in those with infantile CLN1 disease. A large variability in both age at death and disease duration was observed: patients with CLN1 had a longer lifespan (median 8 years 6 months, IQR 4 years 6 months–16 years 4 months) than children with I‐CLN10 (median 6 years 4 months, IQR 2 years 6 months–10 years) (Figure 4a,b).

#### LINCL group

The LINCL group (including patients with classic CLN2 disease and six variant forms, vLINCL) represented the largest cohort in our study (*n* = 101 children). The median age at onset was 3 years 6 months, although children with variants in *CLN1* and *CLN10* had the earliest disease onset. The median age at death was 14 years. A bimodal distribution of age at death in patients with LINCL is represented by the violin plot. The left part of the plot related to a vast majority of patients with CLN2, whose median age at death was 9 years. Conversely, the right part accounted for children with vLINCL, whose median age at death was increased twofold. The IQR deviation of both age at death and disease duration for children with CLN2 was similar (compared with vLINCL) (Figure 4c,d).

#### JNCL group

The JNCL group included 38 patients. Two‐thirds of them were with CLN3 disease, the remainder with juvenile variants (vJNCL) of other NCL forms (CLN1, CLN2, CLN5, CLN6 diseases). The median age at onset was 7 years. The earliest age at onset was observed in patients with CLN3 disease and in three patients with vJ‐CLN2 diseases; the oldest age at onset was seen in four patients with vJ‐CLN6 and one with vJ‐CLN5 whose clinical manifestations started at pubertal age. The median age at death was 29 years; that for patients with CLN3 was 30 years, with an IQR of about 10 years; both median age at death and IQR of the vJNCL forms were similar. The median disease duration for the group (including censored patients) was 23 years 4 months; the IQR of the group, of CLN3 disease, and of vJNCL forms was unchanged. The overall distributions of the group and its components are depicted by the patterns of the violin plots (Figure 4e,f).

### Genetics

#### Variant analysis

Genetic investigation of the CLNet patients identified 111 germline mutations in nine NCL genes: five variants belonging to three NCL genes were shared among different phenotypes (*CLN1*, *CLN2*, *CLN5*). Missense mutations were the most represented of the whole cohort (46.8%) compared with nonsense mutations (36.9%), and they were equally distributed among the groups (Figure S1 and Table S3).

Using the ACMG guidelines, we observed that class 5 and class 4 variants accounted for 79.3% of the whole cohort; VUS (class 3) scored 17.1%; and benign and likely benign (classes 1 and 2) were 3.6%. The percentage of both class 5 and class 4 variants was prevalent in the LINCL group (84.2%). Class 3 variants were more represented in the INCL and JNCL groups (27.3% and 20.8% respectively) and they were clustered within some genes (namely *CLN10* and *CLN6*; Figure S1 and Table S3). Most nonsense mutations and deletions were categorized as class 5 variants; missense mutations and mutations leading to splice defects were categorized as class 5 to class 1 variants.

#### Genotype analysis

**FIGURE 5:**
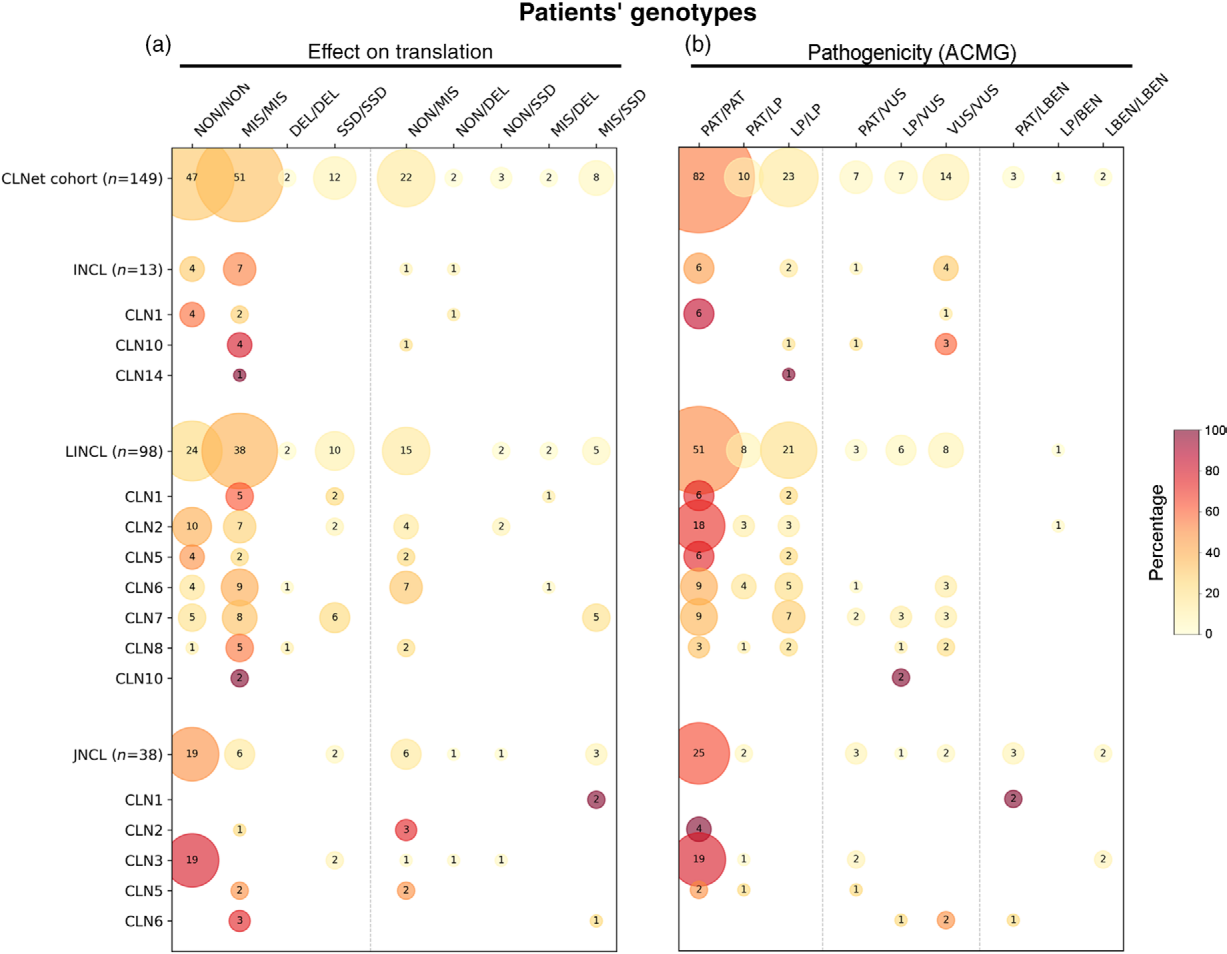
Bubble chart of genotype distribution classified according to the molecular features (effects on the translation of messenger RNA into protein) and ACMG pathogenicity evidence of CLNet cohort genotypes. The bubble size is proportional to the numbers of genotypes (reported inside the bubbles) which were identified in the different groups, whereas the colour gradient represents the relative percentage (ranging from 0% in white to 100% in dark red) in each group or form (see also Table S5). (a) Genotypes carrying either bi‐allelic nonsense or missense mutations were equally distributed among the NCL groups and forms, the former being more represented in *CLN1*, *CLN2*, *LI‐CLN5*, and *CLN3* genotypes, the latter in *LI‐CLN10*, *LI‐CLN1*, and *LI‐CLN8*. The percentage of genotypes carrying bi‐allelic splice site defects was highest in LI‐CLN1 an LI‐CLN7. (b) Following ACMG categorization, almost a half of the CLNet genotypes carried two class 5 (pathogenic) variants; these genotypes were highly represented in patients with CLN2, CLN3, and LI‐CLN5. ACMG tiers: PAT, pathogenic (class 5); LP, likely pathogenic (class 4); VUS, variant of uncertain significance (class 3); LBEN, likely benign (class 2); BEN, benign (class 1). Abbreviations: ACMG, American College of Medical Genetics; CLNet, Italian network of neuronal ceroid lipofuscinoses; DEL, deletion; INCL, infantile neuronal ceroid lipofuscinosis; JNCL, juvenile neuronal ceroid lipofuscinosis; LINCL, late infantile neuronal ceroid lipofuscinosis; MIS, missense mutation; NCL, neuronal ceroid lipofuscinosis; NON, nonsense mutation; SSD, splice site defect.

Bi‐allelic missense and nonsense mutations were the most represented genotypes in the whole cohort (34.2% and 31.5% respectively). In the INCL group the percentage of bi‐allelic missense mutations was about twofold higher than nonsense ones (53.8% vs. 30.8%); the opposite trend was seen in JNCL where bi‐allelic nonsense mutations were prevalent (Figure 5a and Table S4).

According to ACMG guidelines, class 5 and class 4 variants accounted for 89.2% of the whole cohort (both as homozygous and heterozygous compounds). Similar percentages were detected in the LINCL (91.8%) and JNCL (89.5%) groups, whereas lower percentages occurred in those with INCL (69.3%). Class 3 variants (VUS) accounted for 18.8% of the whole cohort genotypes, often in compound heterozygosity with a class 4–5 variant. Bi‐allelic VUS were prevalent in the INCL group (Figure 5b and Table S5).

#### Phenotypic heterogeneity

**FIGURE 6:**
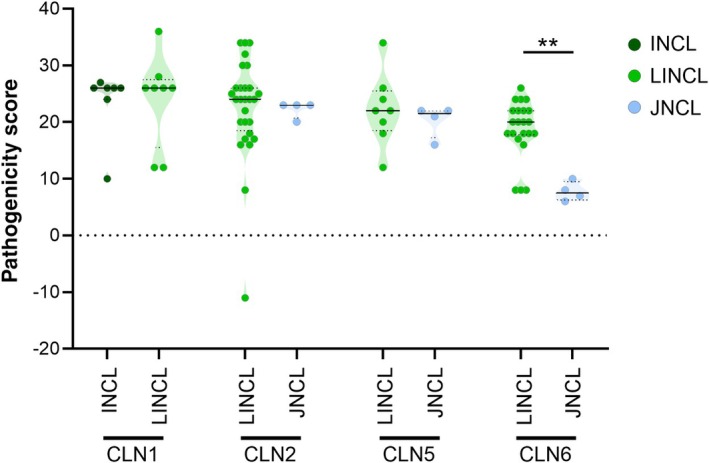
Scatterplot representations of pathogenicity score of *CLN1*, *CLN2*, *CLN5*, and *CLN6* genotypes belonging to different NCL groups (according to American College of Medical Genetics guidelines). A meaningful difference between LINCL and JNCL genotype scores is observed for *CLN6* (*n* = 22 and *n* = 4 respectively). No differences were found for other NCL forms. Mann–Whitney *U* test; **p < 0.01. Black horizontal lines and dotted lines represent the median and IQR respectively. Abbreviations: INCL, infantile neuronal ceroid lipofuscinosis; JNCL, juvenile neuronal ceroid lipofuscinosis; LINCL, late infantile neuronal ceroid lipofuscinosis; NCL, neuronal ceroid lipofuscinosis.

To evaluate whether changes in the genotypes' profiles might be associated with different age‐related disease outcomes, we focused on four NCL genes (*CLN1*, *CLN2*, *CLN5*, *CLN6*) that give origin to NCL forms with different ages at onset and disease durations. Meaningful differences of pathogenicity score of genotypes were observed for patients with mutations in *CLN6* only. Higher scores were associated with the variant late infantile phenotype compared with the variant juvenile one, which showed an increased percentage of VUS, both as homozygous and heterozygous compounds (Figure 6).

## DISCUSSION

Results from this study have provided some general information correlating the clinical course and the role variant pathogenicity with phenotype expression in a large group of patients with childhood‐onset NCL. Cumulative data have shown the median lifespan (17 years) is short in the patients enrolled in the Italian CLNet cohort, whose phenotypes were associated with mutations in eight NCL genes. The strong correlation between age at onset and age at death observed in each NCL age group (Figure 1c) indicates the primary role of the age at clinical onset in determining the disease duration and fatal outcome. These findings seemed to be homogeneously present among the three examined groups of patients with NCL as sustained by the skewed shape of the survival curves (Figure 2). Such homogeneity was partly present when considering the single disease forms of each group. The earliest median age at death, shortest disease duration, narrow IQR values, and rapid decline of the survival rate observed only in classic CLN2 disease were aligned with the clinical features of this condition of late infantile onset.^13^ Children's care might benefit from the increased awareness of the correlation between age at onset and age at death. Efforts to shorten the time for a diagnosis to be achieved, particularly for the early‐onset phenotypes, are mandatory, notably if targeted treatments are (or will be) available. Likewise, recognition of the time course of disease evolution might assist overall home care, aimed at both sustaining neurological and cognitive functions and preventing secondary effects of the neurological impairment.

Phenotypic heterogeneity is quite common in NCLs^8^ and the results of this study acknowledge that. High percentages of genotypes carrying pathogenic variants were distributed among the cohort groups to the same degree, and the identification of pathogenic variants in the same gene did not always anticipate the severe or milder clinical consequences they could lead to. The majority of severe phenotypes (in terms of age at onset and age at death) were associated with mutated genes coding for lysosomal hydrolase (*CLN10*, *CLN1*, and *CLN2*) (Figure 3), but not necessarily linked to class 5 and 4 variants in the related genes. It is interesting to note the prevalent role of age at onset in determining the phenotype as opposed to the genetic profile and the gene product: in late‐onset vJNCL, patients' pathogenic variants of *CLN10*, *CLN1*, and *CLN2* were associated with a milder phenotype characterized by slowly progressive course and prolonged survival. Meaningful changes of variant pathogenicity scores related to the phenotype were detected in a few patients with mutations in *CLN6* who had higher scores of the late infantile phenotype compared with the juvenile one (Figure 6).

To improve the characterization of phenotypic heterogeneity, natural‐history studies of large cohorts are recommended, including scored genetic variants combined with much‐needed fluid biomarkers. We emphasize that the characteristics of the socioeconomic setting of a country also have to be considered, because differences in the quality and efficacy of health systems may influence the clinical conditions and apparently interfere with phenotype expression. The combined analysis might provide elements that might score (or underscore) the influence of variants and genotypes on the clinical course and outcome of diseases associated with NCL genes. To our knowledge, there are no reported studies that have matched the investigated clinical features (age at onset, age at death, disease duration) with variant pathogenicity in NCLs. Therefore, broad international studies are needed to test at molecular levels (variants and genotypes) the differences and similarities detected when matching the age at onset and lifespan of specific NCL forms from this study with other reports (Table 2).^13^, ^14^, ^15^, ^16^, ^17^, ^18^

**TABLE 2:** Age at onset and age at death of patients with different forms of childhood‐onset NCL as reported from the literature and the present study.

		Age at onset		Age at death		
NCL group	Gene	*n*	Median (IQR)	*n*	Median (IQR)	References
INCL	*CLN1*	7	1 (0:11–1)	4	8:6 (4:6–16:4)	This study
		4	1 (0.9–1.1)	4	10.1 (0.7–10.8)	Moore et al.^18^
		6	8–15 months^a^	3	11–12^a^	Perez‐Poyato et al.^17^
LINCL	*CLN1*	8	2:4 (2:4–3:7)	3	17 (16–17)	This study
		3	3 (2.3–3.3)	3	15.8 (12.7–15.9)	Moore et al.^18^
	*CLN2*	23	3 (2:4–8)	13	9 (9–11)	This study
		9	2.3 (2–2.8)	9	11 (9–13.1)	Moore et al.^18^
			3 (24–38.5)^b^	20	Mean 10 (SD 3.2)^a^	Nickel et al.^13^
		28	2.9 (1.8–3.9)	27	8.6 (4–12.9)	Moore et al.^15^
				7	12 (9–14)	Augestad and Flanders^14^
	*CLN6*	22	4 (3–4)	6	18 (15:10–20:4)	This study
		8	4 (3.0–4.9)	8	11.0 (8.0–14.9)	Moore et al.^15^
	*CLN7*	24	3:10 (2:5–4:11)	9	10 (7–19)	This study
		25	3.5 (2.5–4)	7	12 (7–16)	Kuosi et al.^16^
JNCL	*CLN3*	22	6:6 (5:5–8)	8	30 (25:7–34:10)	This study
		59	5.5 (5–6.5)	59	21.3 (18.2–22.1); 21.6 (19.1–26.8)	Moore et al.^18^
		63	Mean 8.2 (SD 2.3)^a^	63	26 (25–30)	Augestad and Flanders^14^
		3	6.7 (6.5–7.1)	3	18.9 (15.1–23.3)	Moore et al.^15^

*Note*: Unless otherwise specified, age values are reported in years and months for this study and in decimal years for the other studies as originally presented.

^a^ Incomplete data (no IQR or mean SD instead of median).

^b^ Months.

Abbreviations: INCL, infantile neuronal ceroid lipofuscinosis; IQR, interquartile range; JNCL, juvenile neuronal ceroid lipofuscinosis; NCL, neuronal ceroid lipofuscinosis; LINCL, late infantile neuronal ceroid lipofuscinosis; SE, standard error.

The lack of a clear‐cut association between ranked variants and the phenotypes of NCL forms may be not surprising because, on the basis of current knowledge, this is often the case in infantile or childhood‐onset neurodegenerative conditions.^19^ A relatively high number of class 3 variants (VUS) was present, accounting for 17.1% of the whole cohort. The effects that originate from the mutated NCL genes carrying class 3 variants are still only partly known, and the diagnosis based on these variants can be still questionable.^20^, ^21^, ^22^

The apparent discrepancies between disease onset and duration and genotype profile raise the question of which signals acting at selected temporal windows drive the mutated NCL proteins to exert their pathological effect and thereafter determine the timing of cell death, the length of survival, and ultimately the phenotypic manifestations. Comparative functional studies, using selected variants of NCL genes in appropriate settings (e.g. vertebrate models, cell systems, etc.) and designed for the temporal expression of neuronal damage being tested at sequential time points, are recommended. A last remark is worth making. The availability of orphan drugs has made it possible to slow the decline and extend the survival in several lysosomal storage diseases affecting the brain.^23^, ^24^, ^25^, ^26^ A major challenge in considering the efficacy of innovative treatments is whether the decreased rate of clinical decline over longer periods for NCLs, such as for classic CLN2 disease,^27^ is accompanied by improved quality of life as well as prolonged survival of the treated patients, extending the lifespan over those in pretreatment decades.^14^ As such, predictive values of progression combining imaging/fluid/digital biomarkers with scoring of genetic variants and machine learning approaches to systems biology may increase the amount of information and contribute to better evaluation of the impact of new (and future) targeted treatments^28^, ^29^, ^30^ on survival rates.

## FUNDING INFORMATION

The Italian Ministry for Universities and Research; Fondazione Mariani (National Network on Rare Neuropediatric Diseases); Tuscany Region (Bando Ricerca Salute 2018, DEM AGING); Italian Ministry of Health, Ricerca Corrente 2025 and 5x1000; Rete IRCCS‐RIN.

## CONFLICTS OF INTEREST STATEMENT

The authors report no conflict of interest.

## Supporting information


**Appendix S1:** Methods.
**Figure S1:** Molecular features and ACMG pathogenetic classification of variants identified in the CLNet cohort.

**Table S1:** Demographic Data of the CLNet Cohort.

**Table S2:** Clinical Data of the CLNet Cohort.

**Table S3:** Molecular Features of Variants Identified in the CLNet Cohort and Pathogenicty Score.

**Table S4:** Percentage distribution of variants, classified according to the molecular features (types of DNA mutation, effects of mRNA translation into protein) and pathogenicity score.

**Table S5:** Percentage distribution of genotypes, classified according to the molecular features (effects of mRNA translation into protein) and pathogenicity score.

## Data Availability

The data that support the findings of this study are available from the corresponding author upon reasonable request.
