# Supplementary material for: Age at onset and gene variants predict lifespan and disease duration in childhood neuronal ceroid lipofuscinoses
Source: Dev Med Child Neurol. 2025 Jul 24;68(2):276–86. doi: 10.1111/dmcn.16416 (PMC12766547; doi:10.1111/dmcn.16416)
Supplement: Supplementary file 1 — Appendix S1: Methods. Figure S1: Molecular features and ACMG pathogenetic classification of variants identified in the CLNet cohort. [file DMCN-68-276-s001.docx]

**Appendix S1**

**Methods**

**Statistics**

Standard descriptive statistics parameters (mean, standard deviation [STD], median and interquartile range [IQR]) were used to evaluate the AO, AD and DD for the overall NCL cohort as well as for each NCL group and form, including censored patients*.* The distribution as well as the density of clinical data were represented by violin plots. Bubble chart representations were used to depict the percentage distribution of both variants and genotypes, categorized according to either the type of mutations (HGVS nomenclature) or the ACMG pathogenetic scoring system, in the different NCL groups and forms.

The normal distribution of data was assessed by Shapiro-Wilk normality test. Accordingly, non-parametric tests were then used to assess the difference between two (Mann-Whitney) or more groups (Kruskal-Wallis test followed by multiple comparisons test).

Correlation analysis among different variables of the whole cohort and within specific groups of patients (the clinical parameters [AO, AD, DD] and the pathogenicity classes and scores was performed by nonparametric Spearman method. The Rho (ρ) coefficient was used to estimate the strength of the correlation between two variables; *P* values were also considered.

For clinical outcome assessment, Kaplan Meier survival curves were generated considering either the AD or the age of last consultation for censored patients for whom the date of death was unavailable or who were still alive. The statistical difference among the survival curves was assessed by pairwise comparisons of two clinical groups (INCL vs LINCL, INCL vs JNCL and LINCL vs JNCL) using the Log-rank test. To correct for multi-comparisons, the obtained *P* values were considered as significant if lower than the Bonferroni-corrected α-threshold*.*

The statistical analyses and graphical representations (including violin plots and survival curves) were performed using GraphPad Prism 10.0.2.

**
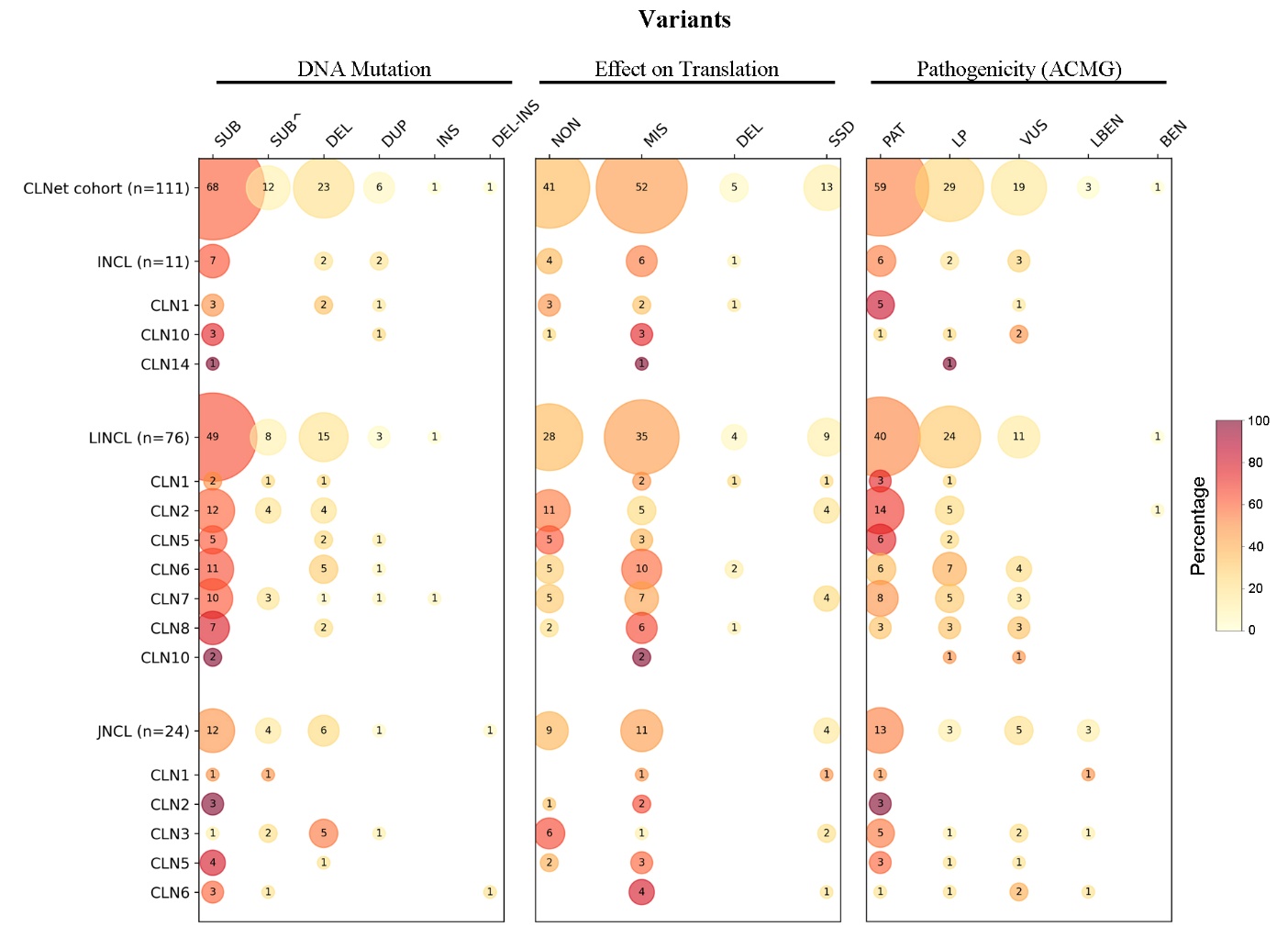
**

**Supporting Figure 1. Molecular features and ACMG pathogenetic classification of variants identified in the CLNet cohort.** Bubble chart representation of variant distribution, classified according to the molecular classifications (types of DNA mutation, effects on the translation of mRNA into protein) and ACMG guidelines. The bubble size is proportional to the numbers of variants (reported inside the bubbles) which were identified in the different groups, whereas the colour gradient represents the relative percentage (ranging from 0% in white to 100% in dark red) within each group or form. Nucleotide substitution (SUB) is the most represented type of mutation in all groups, except for CLN3 in which deletions are the most frequent ones. High percentages of nonsense mutations are observed in CLN1, CLN2, LI-CLN5 and CLN3 whereas missense ones are markedly associated with CLN10 patients (both infantile and late-infantile forms), LI-CLN6, LI-CLN8 and J-CLN5. Highest figures of pathogenic variants are seen in CLN2, CLN3 and LI-CLN5. DNA mutation: SUB, substitution; SUB^ nucleotide substitution in intronic region; DEL, deletion; DUP, duplication, INS, insertion; DEL-INS, deletion with insertion. ACMG classes: PAT, pathogenic (class V); LP, likely pathogenic (class IV); VUS, variant of uncertain significance (class III); LBEN, likely benign (class II); BEN, benign (class I). See Supplementary Table S4 for further information.
